# Tris{2-meth­oxy-6-[(4-methyl­phen­yl)iminiometh­yl]phenolate-κ^2^ *O*,*O*′}tris­(thio­cyanato-κ*N*)europium(III)

**DOI:** 10.1107/S1600536809049125

**Published:** 2009-11-21

**Authors:** Jian-Feng Liu, Jia-Lu Liu, Guo-Liang Zhao

**Affiliations:** a Zhejiang Key Laboratory for Reactive Chemistry on Solid Surfaces, Institute of Physical Chemistry, Zhejiang Normal University, Jinhua, Zhejiang 321004, People’s Republic of China, and College of Chemistry and Life Science, Zhejiang Normal University, Jinhua 321004, Zhejiang, People’s Republic of China

## Abstract

The metal center in the structure of the title compound, [Eu(NCS)_3_(C_15_H_15_NO_2_)_3_], is coordinated by three Schiff base 2-meth­oxy-6-[(4-methyl­phen­yl)iminiometh­yl]phenolate (*L*) ligands and three independent thio­cyanate ions. In the crystal structure, the acidic H atom is located on the Schiff base N atom and hydrogen bonded to the phenolate O atom. The coordination environment of the Eu^III^ ion is nine-coordinate by three chelating methoxy­phenolate pairs of O atoms and three N-atom terminals of the thio­cyanate ions. The compound is isostructural with the Ce^III^ analogue [Liu *et al.* (2009 ▶). *Acta Cryst.* E**65**, m650].

## Related literature

For background to Schiff bases and their applications, see: Liu *et al.* (1997 ▶); Mihara *et al.* (2009 ▶). For related structures, see: Liu *et al.* (2009 ▶); Zhao *et al.* (2007 ▶). For a zigzag chain cadmium(II) complex, see: Li *et al.* (2008 ▶). 
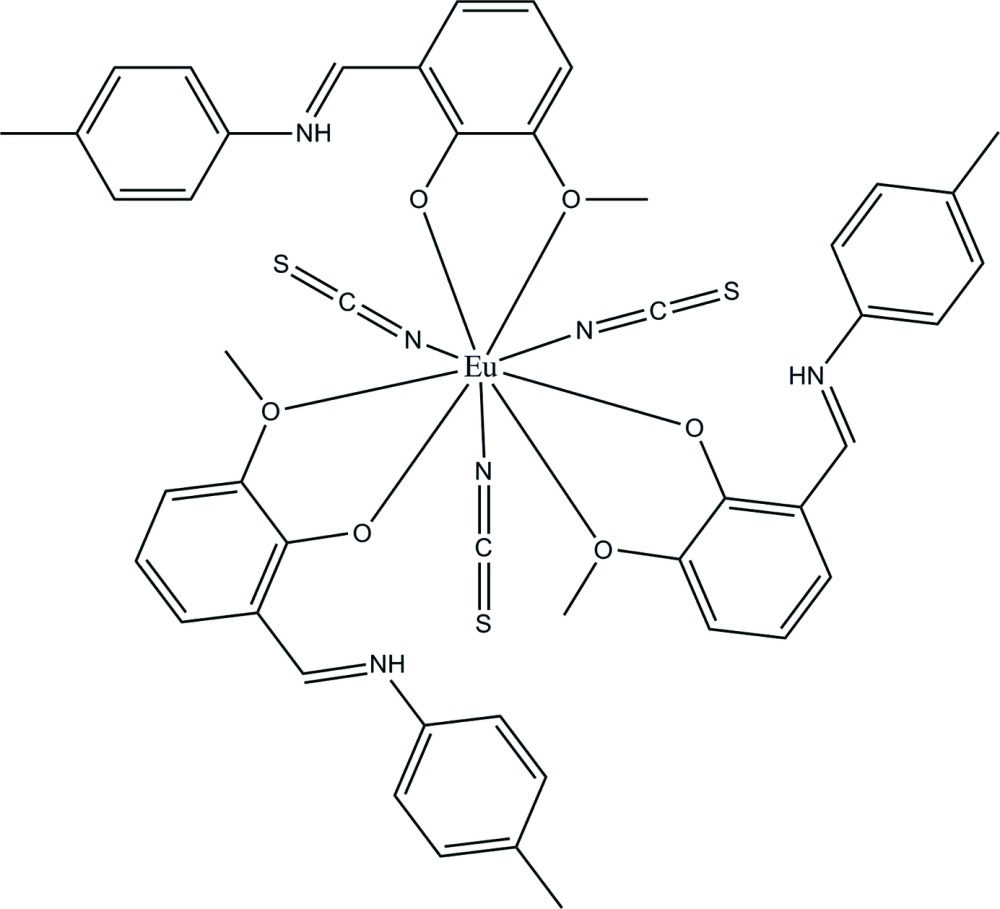


## Experimental

### 

#### Crystal data

[Eu(NCS)_3_(C_15_H_15_NO_2_)_3_]
*M* *_r_* = 1050.04
Monoclinic, 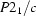

*a* = 16.6445 (2) Å
*b* = 14.2411 (2) Å
*c* = 22.1678 (3) Å
β = 105.912 (1)°
*V* = 5053.23 (12) Å^3^
*Z* = 4
Mo *K*α radiation
μ = 1.42 mm^−1^
*T* = 296 K
0.31 × 0.16 × 0.13 mm

#### Data collection

Bruker APEXII CCD area-detector diffractometer
Absorption correction: multi-scan (*SADABS*; Sheldrick, 1996 ▶) *T*_min_ = 0.757, *T*_max_ = 0.834
37461 measured reflections
8882 independent reflections
6762 reflections with *I* > 2σ(*I*)
*R*_int_ = 0.046

#### Refinement

*R*[*F* ^2^ > 2σ(*F* ^2^)] = 0.045
*wR*(*F* ^2^) = 0.137
*S* = 1.06
8882 reflections
577 parameters
H-atom parameters constrained
Δρ_max_ = 0.87 e Å^−3^
Δρ_min_ = −0.41 e Å^−3^

### 

Data collection: *APEX2* (Bruker, 2006 ▶); cell refinement: *SAINT* (Bruker, 2006 ▶); data reduction: *SAINT*; program(s) used to solve structure: *SHELXS97* (Sheldrick, 2008 ▶); program(s) used to refine structure: *SHELXL97* (Sheldrick, 2008 ▶); molecular graphics: *SHELXTL* (Sheldrick, 2008 ▶); software used to prepare material for publication: *SHELXL97*.

## Supplementary Material

Crystal structure: contains datablocks I, global. DOI: 10.1107/S1600536809049125/zl2242sup1.cif

Structure factors: contains datablocks I. DOI: 10.1107/S1600536809049125/zl2242Isup2.hkl

Additional supplementary materials:  crystallographic information; 3D view; checkCIF report

## Figures and Tables

**Table 1:** Hydrogen-bond geometry (Å, °)

*D*—H⋯*A*	*D*—H	H⋯*A*	*D*⋯*A*	*D*—H⋯*A*
N1—H1*A*⋯O1	0.86	1.89	2.588 (4)	138
N2—H2*A*⋯O3	0.86	1.89	2.580 (4)	137
N3—H3*A*⋯O5	0.86	1.84	2.550 (4)	138

**Fig. 1.**
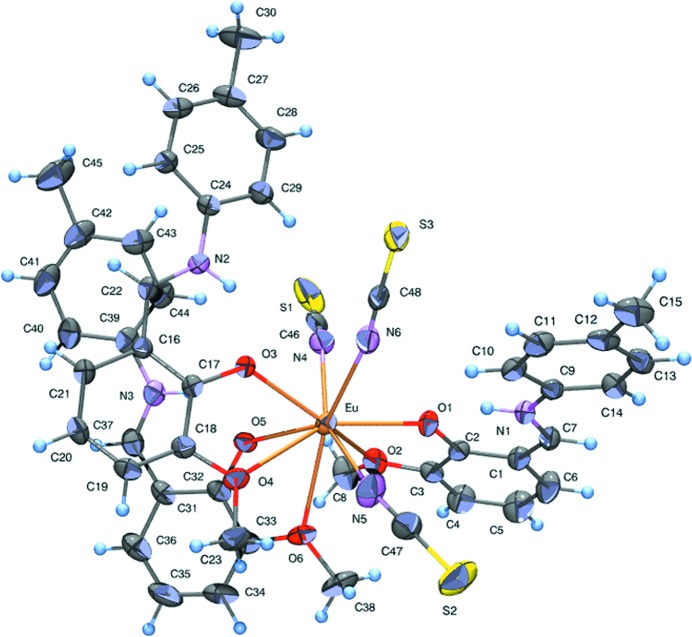
The molecular structure of the title complex, showing the atom-labeling scheme. Displacement ellipsoids are drawn at the 30% probability level.

**Fig. 2.**
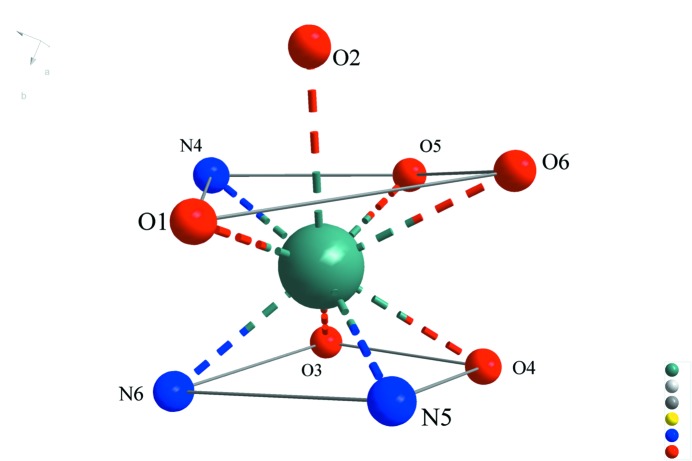
The coordination environment of the europium(III) atom, showing the distorted monocapped square antiprismatic geometry.

## supplementary crystallographic information

### Comment

Rare earth complexes with Schiff ligands derived from *o*-vanillin have been investigated over many years. Liu *et al.* (1997) for example describe structural and chemical properties of vanilline derived complexes, and (Mihara *et al.*, 2009) investigated them as catalytsts for asymmetric α-additions of isocyanides to aldehydes. Over the last years we have been engaged in the syntheses of new analogous Schiff bases derived from *o*-vanillin and their rare metal complexes. In prvious articles we have reported partial results (Zhao *et al.*, 2007; Li *et al.* 2008; Liu *et al.* 2009). Herein, we would like to describe a new Eu^III^ complex.

The structure of the title complex is shown in Fig.1. The structure of [Eu(NCS)_3_.(C_15_H_15_NO_2_)_3_] contains three (H*L*) ligands and three independent thiocyanate ions. The Eu^III^ is nine-coordinated by three *N* terminals from three thiocyanate ions and six O atoms from the H*L* ligands (shown in Fig. 2). The H*L* ligands chelate the Eu^III^ ion with the methoxy O atoms and the deprotonated phenolic hydroxyl O atoms. The Eu—O (phenolic) bonds are 2.773 (3) - 2.839 (3) Å, which are longer than the ones between Eu^III^ and methoxy O atoms (2.376 (3) - 2.418 (3) Å). The Eu—N bonds are 2.512 (5) - 2.566 (5) Å. The Eu—O (phenolic) and Eu—N bonds are shorter than in the isostructural Ce^III^ complex (Liu *et al.* 2009), which can be attributed to the ionic radius decrease from Ce^III^ to Eu^III^ due to the lanthanide contraction. Because of the geometric and chemical environment requirements of the chelating groups, the coordination geometry deviates considerably from that of a distorted capped square antiprismatic geometry (Fig. 2). In the H*L* ligands, the acidic proton has been transferred from the phenolic group to the *N*-imine atom, which is involved in an intramolecular hydrogen bond. All hydrogen bonds are listed in Table 1.

### Experimental

Reagents and solvents were of commercially available quality and were used without further purification. The Schiff base ligand was prepared in a high yield synthesis by condensation of *o*-vanillin and *p*-methylaniline and was recrystallized in ethanol before being used. 1 mmol Eu(NO_3_)_3_ (dissolved in methanol) was added dropwise into a methanol solution with 3 mmol *N*-salicylidene-*p*-toluidine under stirring and the mixture was continuously stirred at room temperature for 8 h to obtain a purplish red solution. The deposit was filtered off and the solution was left standing for slow evaporation. Red crystal of the title compound formed after several days.

### Refinement

The structure was solved by direct methods and successive Fourier difference synthesis and the atom numbering scheme was adopted from the isostructural Ce^III^ complex (Liu *et al.* 2009). The H atoms bonded to C and N atoms were positioned geometrically and refined using a riding model [aliphatic C—H =0.96 Å (*U*_iso_(H) = 1.5*U*_eq_(C)), aromatic C—H = 0.93 Å (*U*_iso_(H) = 1.2*U*_eq_(C)) and N—H = 0.86 Å with *U*_iso_(H) = 1.2*U*_eq_(N)].

### Crystal data

**Table:**

[Eu(NCS)_3_(C_15_H_15_NO_2_)_3_]	*F*(000) = 2136
*M**_r_* = 1050.04	*D*_x_ = 1.380 Mg m^−^^3^
Monoclinic, *P*2_1_/*c*	Mo *K*α radiation, λ = 0.71073 Å
Hall symbol: -P 2ybc	Cell parameters from 7369 reflections
*a* = 16.6445 (2) Å	θ = 2.0–25.0°
*b* = 14.2411 (2) Å	µ = 1.42 mm^−^^1^
*c* = 22.1678 (3) Å	*T* = 296 K
β = 105.912 (1)°	Block, red
*V* = 5053.23 (12) Å^3^	0.31 × 0.16 × 0.13 mm
*Z* = 4	

### Data collection

**Table:**

Bruker APEXII CCD area-detector diffractometer	8882 independent reflections
Radiation source: fine-focus sealed tube	6762 reflections with *I* > 2σ(*I*)
graphite	*R*_int_ = 0.046
phi and ω scans	θ_max_ = 25.0°, θ_min_ = 2.0°
Absorption correction: multi-scan (*SADABS*; Sheldrick, 1996)	*h* = −19→19
*T*_min_ = 0.757, *T*_max_ = 0.834	*k* = −16→16
37461 measured reflections	*l* = −23→26

### Refinement

**Table:**

Refinement on *F*^2^	Primary atom site location: structure-invariant direct methods
Least-squares matrix: full	Secondary atom site location: difference Fourier map
*R*[*F*^2^ > 2σ(*F*^2^)] = 0.045	Hydrogen site location: inferred from neighbouring sites
*wR*(*F*^2^) = 0.137	H-atom parameters constrained
*S* = 1.06	*w* = 1/[σ^2^(*F*_o_^2^) + (0.0857*P*)^2^] where *P* = (*F*_o_^2^ + 2*F*_c_^2^)/3
8882 reflections	(Δ/σ)_max_ = 0.001
577 parameters	Δρ_max_ = 0.87 e Å^−^^3^
0 restraints	Δρ_min_ = −0.41 e Å^−^^3^

### Special details

**Table:**

Geometry. All e.s.d.s (except the e.s.d. in the dihedral angle between two l.s. planes) are estimated using the full covariance matrix. The cell e.s.d.s are taken into account individually in the estimation of e.s.d.s in distances, angles and torsion angles; correlations between e.s.d.s in cell parameters are only used when they are defined by crystal symmetry. An approximate (isotropic) treatment of cell e.s.d.s is used for estimating e.s.d.s involving l.s. planes.
Refinement. Refinement of *F*^2^ against ALL reflections. The weighted *R*-factor *wR* and goodness of fit *S* are based on *F*^2^, conventional *R*-factors *R* are based on *F*, with *F* set to zero for negative *F*^2^. The threshold expression of *F*^2^ > σ(*F*^2^) is used only for calculating *R*-factors(gt) *etc*. and is not relevant to the choice of reflections for refinement. *R*-factors based on *F*^2^ are statistically about twice as large as those based on *F*, and *R*- factors based on ALL data will be even larger.

### Fractional atomic coordinates and isotropic or equivalent isotropic displacement parameters (Å^2^)

**Table:**

	*x*	*y*	*z*	*U*_iso_*/*U*_eq_	
Eu	0.212188 (12)	0.775828 (17)	0.392808 (11)	0.04651 (12)	
C1	−0.0555 (3)	0.7601 (3)	0.4198 (2)	0.0496 (12)	
C2	0.0249 (3)	0.7317 (3)	0.4190 (2)	0.0419 (10)	
C3	0.0421 (3)	0.6343 (3)	0.4173 (2)	0.0482 (11)	
C4	−0.0183 (4)	0.5697 (4)	0.4158 (3)	0.0741 (17)	
H4A	−0.0063	0.5062	0.4143	0.089*	
C5	−0.0979 (4)	0.5975 (4)	0.4164 (3)	0.089 (2)	
H5A	−0.1386	0.5523	0.4152	0.107*	
C6	−0.1172 (3)	0.6901 (4)	0.4186 (3)	0.0768 (18)	
H6A	−0.1707	0.7077	0.4194	0.092*	
C7	−0.0755 (3)	0.8557 (3)	0.4200 (2)	0.0510 (12)	
H7A	−0.1291	0.8720	0.4213	0.061*	
C8	0.1514 (4)	0.5228 (4)	0.4240 (3)	0.0787 (18)	
H8A	0.1088	0.4829	0.4317	0.118*	
H8B	0.2006	0.5189	0.4588	0.118*	
H8C	0.1645	0.5028	0.3864	0.118*	
C9	−0.0370 (2)	1.0207 (3)	0.41823 (19)	0.0417 (10)	
C10	0.0249 (3)	1.0782 (4)	0.4082 (2)	0.0537 (12)	
H10A	0.0731	1.0522	0.4017	0.064*	
C11	0.0150 (3)	1.1736 (4)	0.4081 (3)	0.0618 (13)	
H11A	0.0573	1.2118	0.4017	0.074*	
C12	−0.0553 (4)	1.2144 (3)	0.4170 (2)	0.0585 (14)	
C13	−0.1161 (3)	1.1561 (4)	0.4272 (2)	0.0560 (12)	
H13A	−0.1642	1.1821	0.4339	0.067*	
C14	−0.1071 (3)	1.0589 (3)	0.4279 (2)	0.0512 (11)	
H14A	−0.1488	1.0204	0.4349	0.061*	
C15	−0.0667 (4)	1.3191 (4)	0.4163 (3)	0.0801 (18)	
H15A	−0.0192	1.3490	0.4080	0.120*	
H15B	−0.0719	1.3394	0.4564	0.120*	
H15C	−0.1163	1.3358	0.3842	0.120*	
C16	0.4877 (2)	0.8421 (3)	0.3996 (2)	0.0417 (10)	
C17	0.4005 (2)	0.8307 (3)	0.3755 (2)	0.0401 (10)	
C18	0.3682 (3)	0.8078 (3)	0.3111 (2)	0.0466 (11)	
C19	0.4192 (3)	0.7976 (4)	0.2730 (2)	0.0552 (12)	
H19A	0.3967	0.7839	0.2307	0.066*	
C20	0.5051 (3)	0.8079 (4)	0.2974 (3)	0.0600 (13)	
H20A	0.5395	0.8001	0.2711	0.072*	
C21	0.5395 (3)	0.8290 (4)	0.3585 (2)	0.0561 (13)	
H21A	0.5972	0.8349	0.3738	0.067*	
C22	0.5236 (3)	0.8642 (3)	0.4622 (2)	0.0452 (11)	
H22A	0.5815	0.8699	0.4763	0.054*	
C23	0.2420 (4)	0.7996 (5)	0.2274 (2)	0.082 (2)	
H23A	0.2825	0.8111	0.2047	0.123*	
H23B	0.1996	0.8472	0.2172	0.123*	
H23C	0.2170	0.7391	0.2162	0.123*	
C24	0.5109 (3)	0.8965 (3)	0.5674 (2)	0.0447 (10)	
C25	0.5932 (3)	0.9208 (4)	0.5954 (2)	0.0533 (12)	
H25A	0.6303	0.9285	0.5712	0.064*	
C26	0.6195 (3)	0.9335 (4)	0.6590 (2)	0.0630 (14)	
H26A	0.6749	0.9501	0.6774	0.076*	
C27	0.5675 (4)	0.9228 (4)	0.6963 (3)	0.0688 (15)	
C28	0.4859 (4)	0.9002 (4)	0.6681 (3)	0.0739 (16)	
H28A	0.4488	0.8941	0.6925	0.089*	
C29	0.4578 (3)	0.8862 (4)	0.6044 (2)	0.0635 (14)	
H29A	0.4023	0.8696	0.5864	0.076*	
C30	0.5990 (5)	0.9382 (5)	0.7666 (3)	0.105 (2)	
H30A	0.5541	0.9286	0.7854	0.158*	
H30B	0.6196	1.0012	0.7748	0.158*	
H30C	0.6432	0.8946	0.7842	0.158*	
C31	0.3719 (3)	0.5437 (4)	0.3346 (2)	0.0576 (13)	
C32	0.3006 (3)	0.5925 (3)	0.3394 (2)	0.0496 (11)	
C33	0.2273 (3)	0.5862 (4)	0.2897 (2)	0.0571 (12)	
C34	0.2241 (5)	0.5321 (5)	0.2387 (3)	0.087 (2)	
H34A	0.1750	0.5286	0.2063	0.105*	
C35	0.2944 (6)	0.4814 (6)	0.2347 (3)	0.103 (2)	
H35A	0.2908	0.4432	0.2001	0.123*	
C36	0.3666 (5)	0.4865 (5)	0.2794 (3)	0.0869 (19)	
H36A	0.4132	0.4537	0.2753	0.104*	
C37	0.4482 (3)	0.5515 (4)	0.3816 (3)	0.0591 (13)	
H37A	0.4945	0.5192	0.3767	0.071*	
C38	0.0808 (4)	0.6303 (5)	0.2585 (3)	0.088 (2)	
H38A	0.0801	0.5789	0.2303	0.133*	
H38B	0.0642	0.6868	0.2347	0.133*	
H38C	0.0426	0.6176	0.2830	0.133*	
C39	0.5297 (3)	0.6218 (3)	0.4791 (3)	0.0554 (13)	
C40	0.6085 (3)	0.6166 (4)	0.4702 (3)	0.0703 (15)	
H40A	0.6153	0.5965	0.4320	0.084*	
C41	0.6768 (3)	0.6413 (5)	0.5182 (4)	0.086 (2)	
H41A	0.7296	0.6382	0.5116	0.104*	
C42	0.6700 (3)	0.6700 (4)	0.5745 (4)	0.0795 (18)	
C43	0.5901 (3)	0.6761 (4)	0.5832 (3)	0.0730 (16)	
H43A	0.5838	0.6971	0.6214	0.088*	
C44	0.5208 (3)	0.6515 (4)	0.5359 (3)	0.0631 (14)	
H44A	0.4679	0.6550	0.5424	0.076*	
C45	0.7453 (4)	0.7002 (6)	0.6284 (4)	0.119 (3)	
H45A	0.7957	0.6922	0.6159	0.179*	
H45B	0.7479	0.6622	0.6647	0.179*	
H45C	0.7393	0.7650	0.6383	0.179*	
C46	0.3000 (3)	0.6760 (4)	0.5457 (2)	0.0527 (12)	
C47	0.0442 (4)	0.8630 (5)	0.2737 (3)	0.0781 (17)	
C48	0.2338 (3)	1.0044 (4)	0.4741 (3)	0.0538 (12)	
S1	0.33288 (14)	0.60173 (15)	0.60390 (8)	0.1038 (7)	
S2	−0.05210 (12)	0.86508 (18)	0.23051 (12)	0.1316 (9)	
S3	0.25269 (9)	1.10435 (12)	0.51097 (9)	0.0882 (6)	
N1	−0.0237 (2)	0.9224 (3)	0.41848 (16)	0.0427 (9)	
H1A	0.0254	0.9049	0.4175	0.051*	
N2	0.4802 (2)	0.8774 (2)	0.50229 (16)	0.0418 (8)	
H2A	0.4268	0.8742	0.4876	0.050*	
N3	0.4561 (2)	0.6018 (3)	0.43120 (19)	0.0531 (10)	
H3A	0.4109	0.6263	0.4357	0.064*	
N4	0.2777 (3)	0.7258 (3)	0.5055 (2)	0.0592 (11)	
N5	0.1114 (3)	0.8592 (4)	0.3037 (2)	0.0935 (18)	
N6	0.2185 (2)	0.9354 (3)	0.4477 (2)	0.0652 (12)	
O1	0.08463 (17)	0.7912 (2)	0.42051 (16)	0.0475 (8)	
O2	0.1224 (2)	0.6171 (2)	0.41675 (17)	0.0593 (9)	
O3	0.35011 (16)	0.8406 (2)	0.41093 (13)	0.0443 (7)	
O4	0.28219 (19)	0.8018 (3)	0.29336 (14)	0.0553 (8)	
O5	0.30193 (18)	0.6438 (2)	0.38871 (14)	0.0474 (7)	
O6	0.1634 (2)	0.6416 (3)	0.29926 (15)	0.0611 (9)	

### Atomic displacement parameters (Å^2^)

**Table:**

	*U*^11^	*U*^22^	*U*^33^	*U*^12^	*U*^13^	*U*^23^
Eu	0.03922 (15)	0.05006 (19)	0.04699 (18)	0.00313 (9)	0.00630 (11)	0.00323 (11)
C1	0.046 (2)	0.042 (3)	0.062 (3)	−0.007 (2)	0.017 (2)	−0.002 (2)
C2	0.043 (2)	0.039 (3)	0.042 (3)	−0.0024 (19)	0.0084 (19)	0.001 (2)
C3	0.060 (3)	0.037 (3)	0.049 (3)	−0.003 (2)	0.018 (2)	−0.001 (2)
C4	0.075 (4)	0.041 (3)	0.106 (5)	−0.011 (3)	0.023 (3)	−0.005 (3)
C5	0.083 (4)	0.057 (4)	0.131 (6)	−0.032 (3)	0.038 (4)	−0.007 (4)
C6	0.055 (3)	0.058 (3)	0.123 (5)	−0.015 (3)	0.032 (3)	−0.004 (4)
C7	0.041 (2)	0.053 (3)	0.061 (3)	0.000 (2)	0.017 (2)	−0.008 (2)
C8	0.097 (4)	0.040 (3)	0.110 (5)	0.022 (3)	0.045 (4)	0.012 (3)
C9	0.043 (2)	0.043 (3)	0.036 (2)	0.0028 (19)	0.0059 (18)	0.000 (2)
C10	0.045 (2)	0.053 (3)	0.056 (3)	0.000 (2)	0.002 (2)	−0.001 (2)
C11	0.061 (3)	0.048 (3)	0.069 (4)	−0.010 (2)	0.005 (2)	0.002 (3)
C12	0.078 (3)	0.047 (3)	0.038 (3)	0.006 (3)	−0.005 (2)	−0.002 (2)
C13	0.066 (3)	0.048 (3)	0.053 (3)	0.011 (2)	0.015 (2)	−0.003 (2)
C14	0.058 (3)	0.043 (3)	0.054 (3)	0.003 (2)	0.018 (2)	0.003 (2)
C15	0.112 (5)	0.043 (3)	0.074 (4)	0.001 (3)	0.005 (3)	−0.002 (3)
C16	0.042 (2)	0.043 (3)	0.043 (3)	−0.0041 (18)	0.0156 (19)	−0.002 (2)
C17	0.045 (2)	0.034 (2)	0.042 (3)	−0.0011 (18)	0.0140 (19)	0.0045 (19)
C18	0.053 (2)	0.046 (3)	0.041 (3)	−0.005 (2)	0.013 (2)	0.006 (2)
C19	0.071 (3)	0.059 (3)	0.036 (3)	−0.003 (2)	0.015 (2)	0.000 (2)
C20	0.062 (3)	0.074 (4)	0.055 (3)	−0.001 (3)	0.034 (3)	−0.003 (3)
C21	0.044 (2)	0.070 (4)	0.057 (3)	−0.001 (2)	0.019 (2)	−0.005 (3)
C22	0.037 (2)	0.046 (3)	0.054 (3)	−0.0017 (18)	0.015 (2)	0.001 (2)
C23	0.068 (3)	0.139 (6)	0.031 (3)	−0.019 (4)	0.001 (2)	0.018 (3)
C24	0.052 (2)	0.039 (3)	0.042 (3)	0.0001 (19)	0.013 (2)	−0.002 (2)
C25	0.048 (2)	0.058 (3)	0.054 (3)	−0.009 (2)	0.014 (2)	−0.007 (2)
C26	0.069 (3)	0.055 (3)	0.056 (3)	−0.009 (3)	0.002 (3)	−0.010 (3)
C27	0.104 (4)	0.053 (3)	0.047 (3)	−0.013 (3)	0.017 (3)	−0.009 (3)
C28	0.098 (4)	0.083 (4)	0.048 (3)	−0.024 (3)	0.032 (3)	−0.013 (3)
C29	0.064 (3)	0.070 (4)	0.062 (3)	−0.016 (3)	0.025 (3)	−0.016 (3)
C30	0.161 (7)	0.099 (6)	0.050 (4)	−0.034 (5)	0.020 (4)	−0.015 (4)
C31	0.072 (3)	0.053 (3)	0.054 (3)	0.004 (2)	0.027 (3)	−0.007 (2)
C32	0.066 (3)	0.045 (3)	0.041 (3)	−0.001 (2)	0.019 (2)	0.005 (2)
C33	0.067 (3)	0.055 (3)	0.046 (3)	−0.004 (2)	0.009 (2)	0.003 (2)
C34	0.111 (5)	0.091 (5)	0.049 (4)	−0.024 (4)	0.006 (3)	−0.021 (3)
C35	0.135 (6)	0.105 (6)	0.072 (5)	−0.016 (5)	0.034 (5)	−0.044 (4)
C36	0.112 (5)	0.079 (5)	0.079 (5)	0.003 (4)	0.044 (4)	−0.019 (4)
C37	0.065 (3)	0.042 (3)	0.076 (4)	0.014 (2)	0.029 (3)	0.003 (3)
C38	0.071 (4)	0.090 (5)	0.078 (4)	−0.011 (3)	−0.023 (3)	0.007 (4)
C39	0.055 (3)	0.040 (3)	0.067 (3)	0.014 (2)	0.010 (2)	0.011 (2)
C40	0.060 (3)	0.062 (4)	0.091 (4)	0.022 (3)	0.025 (3)	0.005 (3)
C41	0.050 (3)	0.080 (5)	0.121 (6)	0.018 (3)	0.010 (3)	0.005 (4)
C42	0.062 (3)	0.053 (4)	0.104 (5)	0.012 (3)	−0.010 (3)	0.012 (4)
C43	0.080 (4)	0.049 (3)	0.075 (4)	0.010 (3)	−0.005 (3)	0.001 (3)
C44	0.059 (3)	0.049 (3)	0.077 (4)	0.011 (2)	0.013 (3)	0.010 (3)
C45	0.077 (4)	0.100 (5)	0.146 (7)	0.008 (4)	−0.028 (4)	0.000 (5)
C46	0.061 (3)	0.061 (3)	0.037 (3)	0.017 (2)	0.017 (2)	−0.006 (3)
C47	0.084 (4)	0.082 (4)	0.060 (4)	0.028 (3)	0.006 (3)	0.009 (3)
C48	0.036 (2)	0.057 (3)	0.071 (3)	−0.008 (2)	0.020 (2)	−0.007 (3)
S1	0.1488 (16)	0.1137 (15)	0.0626 (10)	0.0676 (13)	0.0518 (10)	0.0386 (10)
S2	0.0849 (12)	0.1219 (17)	0.148 (2)	0.0366 (12)	−0.0365 (12)	−0.0150 (15)
S3	0.0592 (8)	0.0801 (11)	0.1363 (15)	−0.0291 (7)	0.0455 (9)	−0.0507 (11)
N1	0.0374 (17)	0.043 (2)	0.048 (2)	0.0043 (16)	0.0115 (15)	0.0024 (17)
N2	0.0366 (17)	0.043 (2)	0.043 (2)	−0.0041 (15)	0.0067 (15)	−0.0012 (17)
N3	0.049 (2)	0.045 (2)	0.064 (3)	0.0140 (17)	0.0140 (19)	0.004 (2)
N4	0.061 (3)	0.068 (3)	0.048 (3)	0.012 (2)	0.012 (2)	0.009 (2)
N5	0.065 (3)	0.131 (5)	0.076 (4)	0.032 (3)	0.005 (3)	0.038 (3)
N6	0.052 (2)	0.057 (3)	0.093 (3)	−0.004 (2)	0.029 (2)	−0.014 (3)
O1	0.0397 (15)	0.0340 (17)	0.072 (2)	−0.0014 (12)	0.0208 (15)	0.0004 (15)
O2	0.066 (2)	0.0378 (18)	0.076 (2)	0.0080 (16)	0.0216 (17)	0.0007 (17)
O3	0.0363 (14)	0.056 (2)	0.0420 (17)	−0.0025 (13)	0.0138 (13)	−0.0035 (15)
O4	0.0519 (18)	0.073 (2)	0.0360 (18)	−0.0073 (16)	0.0040 (14)	0.0065 (16)
O5	0.0531 (17)	0.0491 (19)	0.0367 (17)	0.0077 (14)	0.0069 (13)	−0.0026 (15)
O6	0.062 (2)	0.070 (2)	0.0397 (19)	−0.0064 (18)	−0.0051 (15)	0.0018 (17)

### Geometric parameters (Å, °)

**Table:**

Eu—O1	2.376 (3)	C23—H23C	0.9600
Eu—O3	2.404 (3)	C24—C29	1.369 (6)
Eu—O5	2.418 (3)	C24—C25	1.383 (6)
Eu—N5	2.512 (5)	C24—N2	1.420 (5)
Eu—N4	2.538 (4)	C25—C26	1.369 (7)
Eu—N6	2.566 (5)	C25—H25A	0.9300
Eu—O6	2.773 (3)	C26—C27	1.359 (7)
Eu—O4	2.786 (3)	C26—H26A	0.9300
Eu—O2	2.839 (3)	C27—C28	1.368 (8)
C1—C2	1.403 (6)	C27—C30	1.519 (8)
C1—C7	1.402 (7)	C28—C29	1.374 (7)
C1—C6	1.427 (7)	C28—H28A	0.9300
C2—O1	1.299 (5)	C29—H29A	0.9300
C2—C3	1.419 (6)	C30—H30A	0.9600
C3—C4	1.356 (7)	C30—H30B	0.9600
C3—O2	1.362 (5)	C30—H30C	0.9600
C4—C5	1.385 (8)	C31—C32	1.405 (7)
C4—H4A	0.9300	C31—C37	1.409 (7)
C5—C6	1.361 (8)	C31—C36	1.452 (8)
C5—H5A	0.9300	C32—O5	1.310 (5)
C6—H6A	0.9300	C32—C33	1.406 (7)
C7—N1	1.289 (6)	C33—C34	1.356 (8)
C7—H7A	0.9300	C33—O6	1.386 (6)
C8—O2	1.422 (6)	C34—C35	1.398 (10)
C8—H8A	0.9600	C34—H34A	0.9300
C8—H8B	0.9600	C35—C36	1.333 (9)
C8—H8C	0.9600	C35—H35A	0.9300
C9—C14	1.357 (6)	C36—H36A	0.9300
C9—C10	1.380 (6)	C37—N3	1.289 (6)
C9—N1	1.417 (6)	C37—H37A	0.9300
C10—C11	1.369 (7)	C38—O6	1.433 (6)
C10—H10A	0.9300	C38—H38A	0.9600
C11—C12	1.369 (8)	C38—H38B	0.9600
C11—H11A	0.9300	C38—H38C	0.9600
C12—C13	1.376 (7)	C39—C44	1.376 (7)
C12—C15	1.503 (7)	C39—C40	1.380 (7)
C13—C14	1.392 (7)	C39—N3	1.413 (6)
C13—H13A	0.9300	C40—C41	1.374 (8)
C14—H14A	0.9300	C40—H40A	0.9300
C15—H15A	0.9600	C41—C42	1.347 (9)
C15—H15B	0.9600	C41—H41A	0.9300
C15—H15C	0.9600	C42—C43	1.399 (9)
C16—C22	1.389 (6)	C42—C45	1.536 (9)
C16—C17	1.413 (6)	C43—C44	1.374 (7)
C16—C21	1.429 (6)	C43—H43A	0.9300
C17—O3	1.305 (5)	C44—H44A	0.9300
C17—C18	1.418 (6)	C45—H45A	0.9600
C18—C19	1.359 (7)	C45—H45B	0.9600
C18—O4	1.380 (5)	C45—H45C	0.9600
C19—C20	1.390 (7)	C46—N4	1.121 (6)
C19—H19A	0.9300	C46—S1	1.641 (6)
C20—C21	1.352 (7)	C47—N5	1.135 (7)
C20—H20A	0.9300	C47—S2	1.626 (6)
C21—H21A	0.9300	C48—N6	1.137 (6)
C22—N2	1.304 (5)	C48—S3	1.628 (6)
C22—H22A	0.9300	N1—H1A	0.8600
C23—O4	1.431 (6)	N2—H2A	0.8600
C23—H23A	0.9600	N3—H3A	0.8600
C23—H23B	0.9600		
			
O1—Eu—O3	143.27 (10)	O4—C23—H23A	109.5
O1—Eu—O5	133.32 (10)	O4—C23—H23B	109.5
O3—Eu—O5	74.53 (10)	H23A—C23—H23B	109.5
O1—Eu—N5	73.04 (14)	O4—C23—H23C	109.5
O3—Eu—N5	110.57 (15)	H23A—C23—H23C	109.5
O5—Eu—N5	128.62 (15)	H23B—C23—H23C	109.5
O1—Eu—N4	86.82 (13)	C29—C24—C25	118.8 (4)
O3—Eu—N4	79.07 (12)	C29—C24—N2	118.4 (4)
O5—Eu—N4	73.48 (12)	C25—C24—N2	122.7 (4)
N5—Eu—N4	157.03 (16)	C26—C25—C24	119.5 (5)
O1—Eu—N6	73.71 (11)	C26—C25—H25A	120.2
O3—Eu—N6	70.59 (11)	C24—C25—H25A	120.2
O5—Eu—N6	139.54 (12)	C27—C26—C25	122.2 (5)
N5—Eu—N6	83.44 (18)	C27—C26—H26A	118.9
N4—Eu—N6	80.30 (15)	C25—C26—H26A	118.9
O1—Eu—O6	99.07 (10)	C26—C27—C28	117.9 (5)
O3—Eu—O6	117.38 (10)	C26—C27—C30	120.6 (6)
O5—Eu—O6	59.55 (10)	C28—C27—C30	121.5 (6)
N5—Eu—O6	75.07 (16)	C27—C28—C29	121.3 (5)
N4—Eu—O6	120.07 (12)	C27—C28—H28A	119.3
N6—Eu—O6	158.50 (13)	C29—C28—H28A	119.3
O1—Eu—O4	142.12 (10)	C24—C29—C28	120.3 (5)
O3—Eu—O4	59.59 (9)	C24—C29—H29A	119.8
O5—Eu—O4	71.15 (11)	C28—C29—H29A	119.8
N5—Eu—O4	69.46 (14)	C27—C30—H30A	109.5
N4—Eu—O4	131.00 (12)	C27—C30—H30B	109.5
N6—Eu—O4	106.60 (12)	H30A—C30—H30B	109.5
O6—Eu—O4	66.42 (10)	C27—C30—H30C	109.5
O1—Eu—O2	58.09 (9)	H30A—C30—H30C	109.5
O3—Eu—O2	143.34 (10)	H30B—C30—H30C	109.5
O5—Eu—O2	75.55 (10)	C32—C31—C37	120.9 (5)
N5—Eu—O2	104.53 (16)	C32—C31—C36	119.0 (5)
N4—Eu—O2	72.46 (13)	C37—C31—C36	120.1 (5)
N6—Eu—O2	124.88 (12)	O5—C32—C31	121.2 (4)
O6—Eu—O2	61.96 (10)	O5—C32—C33	120.2 (4)
O4—Eu—O2	127.55 (10)	C31—C32—C33	118.6 (5)
C2—C1—C7	120.6 (4)	C34—C33—O6	126.5 (5)
C2—C1—C6	118.9 (5)	C34—C33—C32	121.1 (5)
C7—C1—C6	120.6 (5)	O6—C33—C32	112.3 (4)
O1—C2—C1	122.5 (4)	C33—C34—C35	120.2 (6)
O1—C2—C3	118.7 (4)	C33—C34—H34A	119.9
C1—C2—C3	118.8 (4)	C35—C34—H34A	119.9
C4—C3—O2	126.9 (4)	C36—C35—C34	121.7 (6)
C4—C3—C2	120.7 (5)	C36—C35—H35A	119.2
O2—C3—C2	112.4 (4)	C34—C35—H35A	119.2
C3—C4—C5	120.7 (5)	C35—C36—C31	119.3 (6)
C3—C4—H4A	119.7	C35—C36—H36A	120.3
C5—C4—H4A	119.7	C31—C36—H36A	120.3
C6—C5—C4	120.9 (5)	N3—C37—C31	122.3 (4)
C6—C5—H5A	119.6	N3—C37—H37A	118.8
C4—C5—H5A	119.6	C31—C37—H37A	118.8
C5—C6—C1	120.1 (5)	O6—C38—H38A	109.5
C5—C6—H6A	119.9	O6—C38—H38B	109.5
C1—C6—H6A	119.9	H38A—C38—H38B	109.5
N1—C7—C1	123.6 (4)	O6—C38—H38C	109.5
N1—C7—H7A	118.2	H38A—C38—H38C	109.5
C1—C7—H7A	118.2	H38B—C38—H38C	109.5
O2—C8—H8A	109.5	C44—C39—C40	119.5 (5)
O2—C8—H8B	109.5	C44—C39—N3	117.4 (4)
H8A—C8—H8B	109.5	C40—C39—N3	123.0 (5)
O2—C8—H8C	109.5	C41—C40—C39	119.5 (6)
H8A—C8—H8C	109.5	C41—C40—H40A	120.2
H8B—C8—H8C	109.5	C39—C40—H40A	120.2
C14—C9—C10	119.9 (4)	C42—C41—C40	122.2 (6)
C14—C9—N1	122.4 (4)	C42—C41—H41A	118.9
C10—C9—N1	117.7 (4)	C40—C41—H41A	118.9
C11—C10—C9	119.7 (5)	C41—C42—C43	118.1 (5)
C11—C10—H10A	120.2	C41—C42—C45	123.2 (7)
C9—C10—H10A	120.2	C43—C42—C45	118.6 (7)
C12—C11—C10	121.9 (5)	C44—C43—C42	120.7 (6)
C12—C11—H11A	119.1	C44—C43—H43A	119.6
C10—C11—H11A	119.1	C42—C43—H43A	119.6
C11—C12—C13	117.7 (5)	C43—C44—C39	119.8 (5)
C11—C12—C15	122.1 (5)	C43—C44—H44A	120.1
C13—C12—C15	120.3 (5)	C39—C44—H44A	120.1
C12—C13—C14	121.3 (5)	C42—C45—H45A	109.5
C12—C13—H13A	119.3	C42—C45—H45B	109.5
C14—C13—H13A	119.3	H45A—C45—H45B	109.5
C9—C14—C13	119.5 (5)	C42—C45—H45C	109.5
C9—C14—H14A	120.3	H45A—C45—H45C	109.5
C13—C14—H14A	120.3	H45B—C45—H45C	109.5
C12—C15—H15A	109.5	N4—C46—S1	179.1 (5)
C12—C15—H15B	109.5	N5—C47—S2	178.2 (7)
H15A—C15—H15B	109.5	N6—C48—S3	178.2 (5)
C12—C15—H15C	109.5	C7—N1—C9	128.5 (4)
H15A—C15—H15C	109.5	C7—N1—H1A	115.7
H15B—C15—H15C	109.5	C9—N1—H1A	115.7
C22—C16—C17	121.4 (4)	C22—N2—C24	127.4 (4)
C22—C16—C21	119.8 (4)	C22—N2—H2A	116.3
C17—C16—C21	118.8 (4)	C24—N2—H2A	116.3
O3—C17—C16	121.5 (4)	C37—N3—C39	128.1 (4)
O3—C17—C18	120.2 (4)	C37—N3—H3A	115.9
C16—C17—C18	118.3 (4)	C39—N3—H3A	115.9
C19—C18—O4	126.3 (4)	C46—N4—Eu	157.0 (4)
C19—C18—C17	121.4 (4)	C47—N5—Eu	145.2 (5)
O4—C18—C17	112.2 (4)	C48—N6—Eu	169.8 (4)
C18—C19—C20	119.8 (5)	C2—O1—Eu	131.6 (3)
C18—C19—H19A	120.1	C3—O2—C8	118.3 (4)
C20—C19—H19A	120.1	C3—O2—Eu	115.3 (3)
C21—C20—C19	121.5 (5)	C8—O2—Eu	126.3 (3)
C21—C20—H20A	119.2	C17—O3—Eu	126.8 (3)
C19—C20—H20A	119.2	C18—O4—C23	116.7 (4)
C20—C21—C16	120.2 (4)	C18—O4—Eu	114.2 (3)
C20—C21—H21A	119.9	C23—O4—Eu	128.7 (3)
C16—C21—H21A	119.9	C32—O5—Eu	126.6 (3)
N2—C22—C16	123.1 (4)	C33—O6—C38	118.9 (4)
N2—C22—H22A	118.4	C33—O6—Eu	115.3 (3)
C16—C22—H22A	118.5	C38—O6—Eu	125.7 (4)
			
C7—C1—C2—O1	2.4 (8)	O4—Eu—N5—C47	135.1 (10)
C6—C1—C2—O1	−179.4 (5)	O2—Eu—N5—C47	10.0 (10)
C7—C1—C2—C3	−178.5 (4)	O1—Eu—N6—C48	154 (3)
C6—C1—C2—C3	−0.3 (8)	O3—Eu—N6—C48	−17 (3)
O1—C2—C3—C4	179.9 (5)	O5—Eu—N6—C48	15 (3)
C1—C2—C3—C4	0.8 (8)	N5—Eu—N6—C48	−132 (3)
O1—C2—C3—O2	−0.8 (6)	N4—Eu—N6—C48	65 (3)
C1—C2—C3—O2	−179.9 (4)	O6—Eu—N6—C48	−133 (2)
O2—C3—C4—C5	−179.8 (6)	O4—Eu—N6—C48	−65 (3)
C2—C3—C4—C5	−0.6 (9)	O2—Eu—N6—C48	125 (3)
C3—C4—C5—C6	−0.1 (11)	C1—C2—O1—Eu	−161.0 (3)
C4—C5—C6—C1	0.6 (11)	C3—C2—O1—Eu	19.9 (6)
C2—C1—C6—C5	−0.4 (9)	O3—Eu—O1—C2	−156.8 (3)
C7—C1—C6—C5	177.8 (6)	O5—Eu—O1—C2	−26.2 (4)
C2—C1—C7—N1	1.1 (8)	N5—Eu—O1—C2	101.3 (4)
C6—C1—C7—N1	−177.1 (5)	N4—Eu—O1—C2	−89.9 (4)
C14—C9—C10—C11	−0.1 (7)	N6—Eu—O1—C2	−170.7 (4)
N1—C9—C10—C11	−179.5 (4)	O6—Eu—O1—C2	30.1 (4)
C9—C10—C11—C12	−0.6 (8)	O4—Eu—O1—C2	93.1 (4)
C10—C11—C12—C13	1.0 (8)	O2—Eu—O1—C2	−18.6 (4)
C10—C11—C12—C15	−179.5 (5)	C4—C3—O2—C8	−9.5 (8)
C11—C12—C13—C14	−0.7 (8)	C2—C3—O2—C8	171.3 (4)
C15—C12—C13—C14	179.8 (5)	C4—C3—O2—Eu	166.7 (5)
C10—C9—C14—C13	0.4 (7)	C2—C3—O2—Eu	−12.6 (5)
N1—C9—C14—C13	179.8 (4)	O1—Eu—O2—C3	15.0 (3)
C12—C13—C14—C9	0.0 (8)	O3—Eu—O2—C3	153.1 (3)
C22—C16—C17—O3	0.2 (7)	O5—Eu—O2—C3	−170.7 (3)
C21—C16—C17—O3	179.2 (4)	N5—Eu—O2—C3	−43.9 (3)
C22—C16—C17—C18	−179.8 (4)	N4—Eu—O2—C3	112.4 (3)
C21—C16—C17—C18	−0.8 (6)	N6—Eu—O2—C3	48.2 (4)
O3—C17—C18—C19	179.5 (4)	O6—Eu—O2—C3	−107.8 (3)
C16—C17—C18—C19	−0.5 (7)	O4—Eu—O2—C3	−119.0 (3)
O3—C17—C18—O4	2.1 (6)	O1—Eu—O2—C8	−169.3 (5)
C16—C17—C18—O4	−178.0 (4)	O3—Eu—O2—C8	−31.1 (5)
O4—C18—C19—C20	178.4 (5)	O5—Eu—O2—C8	5.0 (4)
C17—C18—C19—C20	1.3 (8)	N5—Eu—O2—C8	131.8 (4)
C18—C19—C20—C21	−0.8 (9)	N4—Eu—O2—C8	−71.9 (4)
C19—C20—C21—C16	−0.6 (9)	N6—Eu—O2—C8	−136.1 (4)
C22—C16—C21—C20	−179.6 (5)	O6—Eu—O2—C8	67.9 (4)
C17—C16—C21—C20	1.3 (8)	O4—Eu—O2—C8	56.8 (4)
C17—C16—C22—N2	−1.2 (7)	C16—C17—O3—Eu	−157.3 (3)
C21—C16—C22—N2	179.8 (4)	C18—C17—O3—Eu	22.6 (6)
C29—C24—C25—C26	0.3 (7)	O1—Eu—O3—C17	−160.9 (3)
N2—C24—C25—C26	−176.6 (4)	O5—Eu—O3—C17	54.1 (3)
C24—C25—C26—C27	0.2 (8)	N5—Eu—O3—C17	−71.9 (4)
C25—C26—C27—C28	−1.1 (9)	N4—Eu—O3—C17	129.8 (3)
C25—C26—C27—C30	−179.6 (6)	N6—Eu—O3—C17	−146.8 (4)
C26—C27—C28—C29	1.7 (9)	O6—Eu—O3—C17	11.4 (4)
C30—C27—C28—C29	−179.9 (6)	O4—Eu—O3—C17	−22.9 (3)
C25—C24—C29—C28	0.2 (8)	O2—Eu—O3—C17	90.4 (3)
N2—C24—C29—C28	177.3 (5)	C19—C18—O4—C23	−10.6 (8)
C27—C28—C29—C24	−1.3 (9)	C17—C18—O4—C23	166.7 (5)
C37—C31—C32—O5	−2.5 (8)	C19—C18—O4—Eu	163.0 (4)
C36—C31—C32—O5	178.9 (5)	C17—C18—O4—Eu	−19.7 (5)
C37—C31—C32—C33	176.7 (5)	O1—Eu—O4—C18	160.7 (3)
C36—C31—C32—C33	−1.8 (7)	O3—Eu—O4—C18	21.3 (3)
O5—C32—C33—C34	−178.8 (5)	O5—Eu—O4—C18	−61.4 (3)
C31—C32—C33—C34	1.9 (8)	N5—Eu—O4—C18	152.2 (4)
O5—C32—C33—O6	2.5 (6)	N4—Eu—O4—C18	−15.5 (4)
C31—C32—C33—O6	−176.8 (4)	N6—Eu—O4—C18	76.0 (3)
O6—C33—C34—C35	178.4 (6)	O6—Eu—O4—C18	−125.6 (3)
C32—C33—C34—C35	−0.1 (9)	O2—Eu—O4—C18	−114.9 (3)
C33—C34—C35—C36	−1.9 (12)	O1—Eu—O4—C23	−26.6 (5)
C34—C35—C36—C31	1.9 (12)	O3—Eu—O4—C23	−166.0 (5)
C32—C31—C36—C35	−0.1 (9)	O5—Eu—O4—C23	111.3 (5)
C37—C31—C36—C35	−178.6 (6)	N5—Eu—O4—C23	−35.1 (5)
C32—C31—C37—N3	1.1 (8)	N4—Eu—O4—C23	157.2 (5)
C36—C31—C37—N3	179.6 (5)	N6—Eu—O4—C23	−111.3 (5)
C44—C39—C40—C41	−0.1 (8)	O6—Eu—O4—C23	47.1 (5)
N3—C39—C40—C41	176.9 (5)	O2—Eu—O4—C23	57.8 (5)
C39—C40—C41—C42	0.7 (9)	C31—C32—O5—Eu	154.9 (4)
C40—C41—C42—C43	−1.3 (10)	C33—C32—O5—Eu	−24.3 (6)
C40—C41—C42—C45	−178.6 (6)	O1—Eu—O5—C32	94.5 (4)
C41—C42—C43—C44	1.4 (9)	O3—Eu—O5—C32	−113.6 (3)
C45—C42—C43—C44	178.8 (6)	N5—Eu—O5—C32	−9.4 (4)
C42—C43—C44—C39	−0.9 (8)	N4—Eu—O5—C32	163.5 (4)
C40—C39—C44—C43	0.2 (8)	N6—Eu—O5—C32	−144.7 (3)
N3—C39—C44—C43	−177.0 (5)	O6—Eu—O5—C32	22.2 (3)
C1—C7—N1—C9	179.7 (4)	O4—Eu—O5—C32	−51.0 (3)
C14—C9—N1—C7	8.8 (7)	O2—Eu—O5—C32	87.9 (3)
C10—C9—N1—C7	−171.8 (5)	C34—C33—O6—C38	12.3 (8)
C16—C22—N2—C24	177.3 (4)	C32—C33—O6—C38	−169.1 (5)
C29—C24—N2—C22	−164.6 (5)	C34—C33—O6—Eu	−163.4 (5)
C25—C24—N2—C22	12.3 (7)	C32—C33—O6—Eu	15.2 (5)
C31—C37—N3—C39	−174.4 (5)	O1—Eu—O6—C33	−153.9 (3)
C44—C39—N3—C37	−160.3 (5)	O3—Eu—O6—C33	30.8 (3)
C40—C39—N3—C37	22.6 (8)	O5—Eu—O6—C33	−18.5 (3)
O1—Eu—N4—C46	94.1 (10)	N5—Eu—O6—C33	136.5 (3)
O3—Eu—N4—C46	−120.0 (10)	N4—Eu—O6—C33	−62.3 (3)
O5—Eu—N4—C46	−43.0 (10)	N6—Eu—O6—C33	137.8 (4)
N5—Eu—N4—C46	122.6 (10)	O4—Eu—O6—C33	62.8 (3)
N6—Eu—N4—C46	168.1 (11)	O2—Eu—O6—C33	−107.6 (3)
O6—Eu—N4—C46	−4.5 (11)	O1—Eu—O6—C38	30.8 (4)
O4—Eu—N4—C46	−88.3 (10)	O3—Eu—O6—C38	−144.6 (4)
O2—Eu—N4—C46	36.6 (10)	O5—Eu—O6—C38	166.2 (4)
O1—Eu—N5—C47	−39.5 (9)	N5—Eu—O6—C38	−38.8 (4)
O3—Eu—N5—C47	179.2 (9)	N4—Eu—O6—C38	122.4 (4)
O5—Eu—N5—C47	92.9 (9)	N6—Eu—O6—C38	−37.5 (6)
N4—Eu—N5—C47	−69.3 (12)	O4—Eu—O6—C38	−112.6 (4)
N6—Eu—N5—C47	−114.4 (10)	O2—Eu—O6—C38	77.0 (4)
O6—Eu—N5—C47	65.1 (9)		

### Hydrogen-bond geometry (Å, °)

**Table:**

*D*—H···*A*	*D*—H	H···*A*	*D*···*A*	*D*—H···*A*
N1—H1A···O1	0.86	1.89	2.588 (4)	138
N2—H2A···O3	0.86	1.89	2.580 (4)	137
N3—H3A···O5	0.86	1.84	2.550 (4)	138
